# A Prospective, Observational Study on the Application of Ultra-Wide-Field Angiography in the Evaluation and Management of Patients with Anterior Uveitis

**DOI:** 10.1371/journal.pone.0122749

**Published:** 2015-03-27

**Authors:** Ying Chi, Chunying Guo, Yuan Peng, Lijun Qiao, Liu Yang

**Affiliations:** Department of Ophthalmology, Peking University First Hospital, Beijing China; Key Laboratory of Vision Loss and Restoration, Ministry of Education, Beijing, China; Oregon Health &amp; Science University, UNITED STATES

## Abstract

**Purpose:**

Evaluation of peripheral retinal vascular changes in anterior uveitis using ultra-wide-field fluorescein angiography.

**Design:**

A prospective, observational study of a case series of patients diagnosed with anterior uveitis.

**Methods:**

Setting: Clinical observation at an academic medical center. Patient or Study Population: A total of 65 eyes of 33 patients corresponded with the research criteria of anterior uveitis in the opinion of specialists of Peking University First Hospital. Observation Procedures: Patients were diagnosed primarily through clinical examinations and conventional fluorescein angiography. Subsequently, ultra-wide-field fluorescein angiograms were obtained for each patient. Main Outcome Measures: The main outcome was the detection of peripheral retinal changes by ultra-wide-field fluorescein angiography, and how these changes influenced the evaluation and management of the disease.

**Results:**

Peripheral vessel leakage was detected in 27 eyes (42%) with anterior uveitis, of which 15 eyes displayed active inflammation and 12 eyes displayed inactive inflammation. Peripheral vessel leakage was found in seven of eight eyes with cystoid macular edema. Cystoid macular edema was detected in 7 of 27 eyes (26%) with peripheral vessel leakage, whereas 1 of 38 eyes (3%) did not display peripheral vessel leakage (p<0.01). 44.4% of the patients with peripheral vessel leakage had a specific etiology. The relevant treatment strategies were modified based on the results of the ultra-wide-field fluorescein angiography. 12 patients with peripheral vessel leakage and a quiescent anterior segment were added to those receiving topical glucocorticoids, while 3 patients with serious peripheral vessel leakage and an active anterior segment received a sub-Tenon injection of triamcinolone acetonide.

**Conclusions:**

Ultra-wide-field fluorescein angiography was very effective in detecting peripheral retinal vascular pathology in anterior uveitis. The changes found in the periphery were important in the evaluation and management of anterior uveitis.

## Introduction

Peripheral retinal changes are frequently associated with many vision-threatening diseases, including uveitis. Therefore, the evaluation of the retinal periphery is important for the screening, diagnosis, monitoring, and treatment of uveitis. However, conventional angiography offers only a 30−55° degree field of view or up to 96° when using a fixation lamp and rotatable mirror system[1], which does not provide an image of the peripheral retina. Currently, various ultra-wide-field image systems are available for imaging the peripheral retina, which provide 150° or 200° photographic and angiographic views of the fundus. These systems include the Pomerantzeff camera, Retcam, Panoret, Optos, Heidelberg Spectralis noncontact ultra-wide-field module, and Staurenghi 230 SLO contact-lens system. Ultra-wide-field imaging has been demonstrated to be valuable in the evaluation of several retinal pathologies, including diabetes[2–4], retinal-vein occlusions[5–7], uveitis[8–12], retinal vasculitis[13], choroidal metastasis[14, 15], retinal detachment[16, 17], and retinopathy in prematurity[18, 19]. Awareness of the current limitations of wide-field imaging of the retina is essential to ensure the appropriate utilization of these modalities in clinical practice.

The diagnosis of uveitis often requires careful clinical examination of the peripheral retina. Management decisions are frequently made based on the clinical appearance and angiographic behavior of retinal lesions, including of the peripheral area. Several reports on the management of uveitis patients have suggested that ultra-wide-field angiography could prove useful in the clinical evaluation and treatment such patients[8–11]. However, these reports were restricted to intermediate and posterior uveitis[8–11]. To our knowledge, no anterior uveitis-related studies have been reported. The purpose of the present study was to report our preliminary experience using a Heidelberg Spectralis noncontact, ultra-wide-field module in evaluating the peripheral angiographic expression of the retina in anterior uveitis.

## Materials and Methods

We performed a prospective study on anterior uveitis patients who had undergone ultra-wide-field fluorescein angiography in Peking University First Hospital Department of Ophthalmology. The study design complied with the principles of the Declaration of Helsinki, and all of the procedures were approved by the Committee on Human Studies of Peking University First Hospital. Written consent was obtained from the participants to allow the use of their clinical records. Diagnosis of anterior uveitis was primarily made through clinical examination and conventional fluorescein angiography with a field of view of 30−55°. Inflammation activity was evaluated based on the presence of cells in the anterior chamber. Inactive anterior uveitis was defined anterior chamber cells were rare or absent[20]. All patients shown to have inactive disease stopped receiving treatment. The presence of vitreous cells did not feature in the definition of inactive disease. The initial treatment strategy was based on the diagnostic classification and activity of the inflammation. Ultra-wide-field fluorescein angiograms were subsequently obtained to assess how these images had influenced the evidence of both the diagnosis and activity of the inflammation in each patient. We devised the final treatment strategy according to the ultra-wide-field images. The clinical examinations were performed by uveitis specialists. The Heidelberg Spectralis HRA was used to obtain both the conventional fluorescein and the ultra-wide-field fluorescein angiograms during the same visit by changing the lens. Angiography was performed using an intravenous injection of 5 ml of 10% sodium fluorescein after mydriasis. The primary diagnosis, inflammation activity, and treatment strategy were compared with those suggested by ultra-wide-field fluorescein angiography.

## Results

A total of 66 eyes from 33 patients were included. One eye was excluded from the analysis because of poor imaging function. The mean age of the patients was 40.6 ± 15.3yrs.

Peripheral retinal changes were found in 27 of the 65 eyes (42%) of the 33 patients analyzed using ultra-wide-field fluorescein angiography, none of which were identified by other clinical examinations. These changes included peripheral vessel leakage, and hyperfluorescence dot.

Active inflammation (anterior-chamber cell and flare) was detected in 27 of the 65 eyes (42%) of the 33 anterior-uveitis patients through clinical examination and conventional fluorescein angiography, whereas the remaining 38 eyes (58%) were inactive. The time from discontinuation of drug treatment and evaluation ranged from 2 weeks to 6 months (mean, 2.4 months). Each patient underwent ultra-wide-field fluorescein angiography.

**Fig 1:**
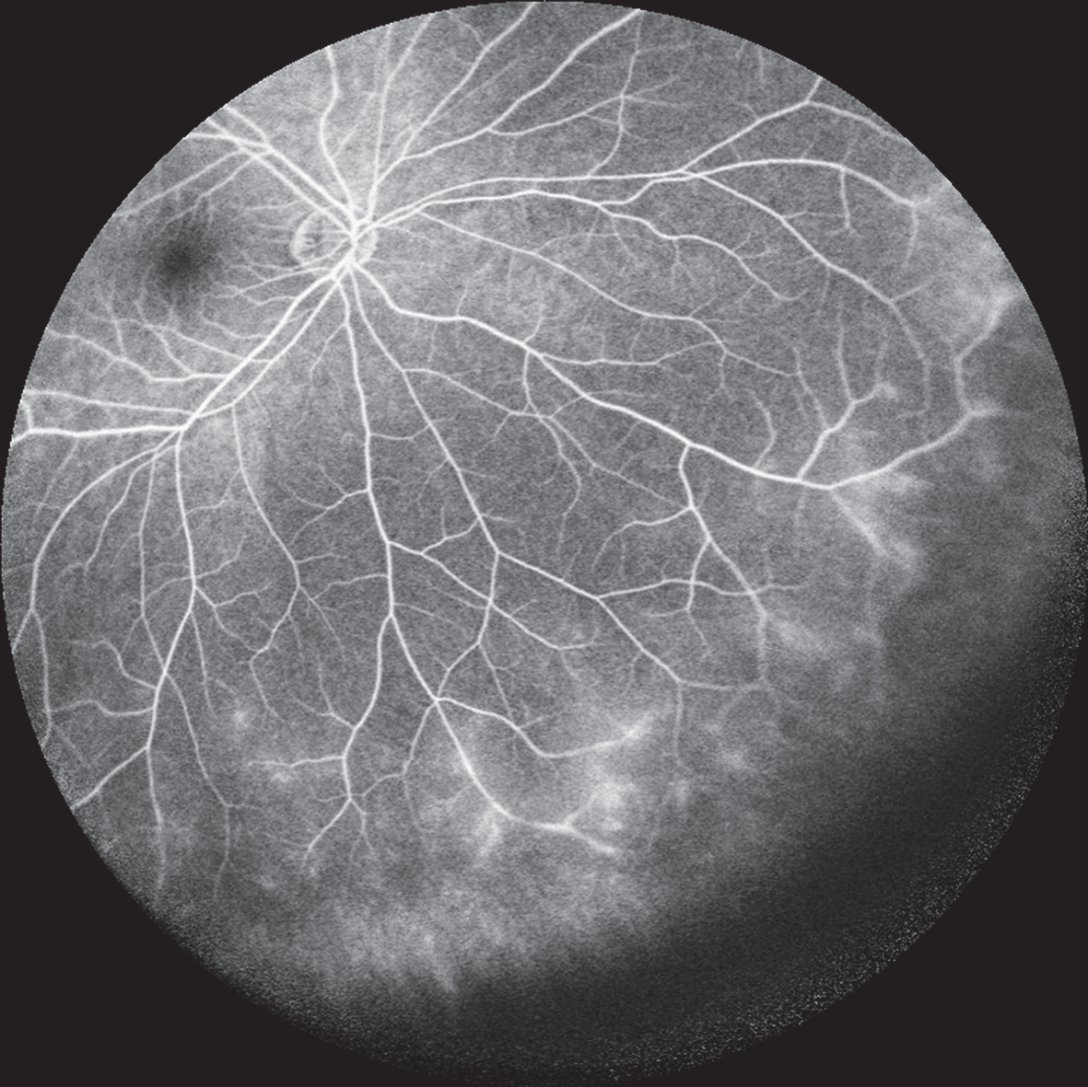
Anterior uveitis with active disease. In a 30-year-old female with active anterior uveitis, peripheral vessel leakage was detected using ultra-wide-field fluorescein angiography.

**Fig 2:**
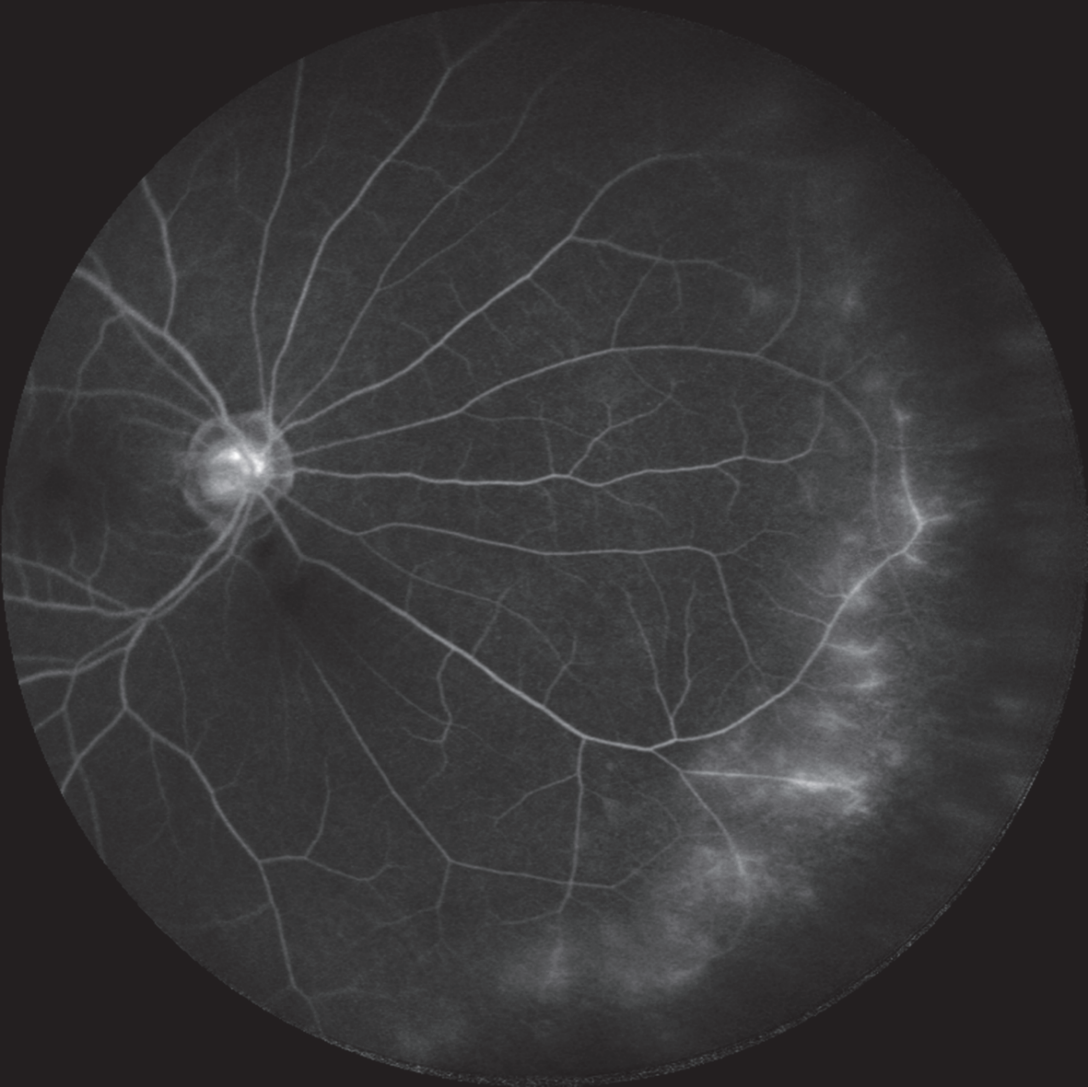
Recurrent anterior uveitis with quiescent disease. A 50-year-old female with recurrent anterior uveitis. The inflammation was quiescent and peripheral vessel leakage was detected.

**Fig 3:**
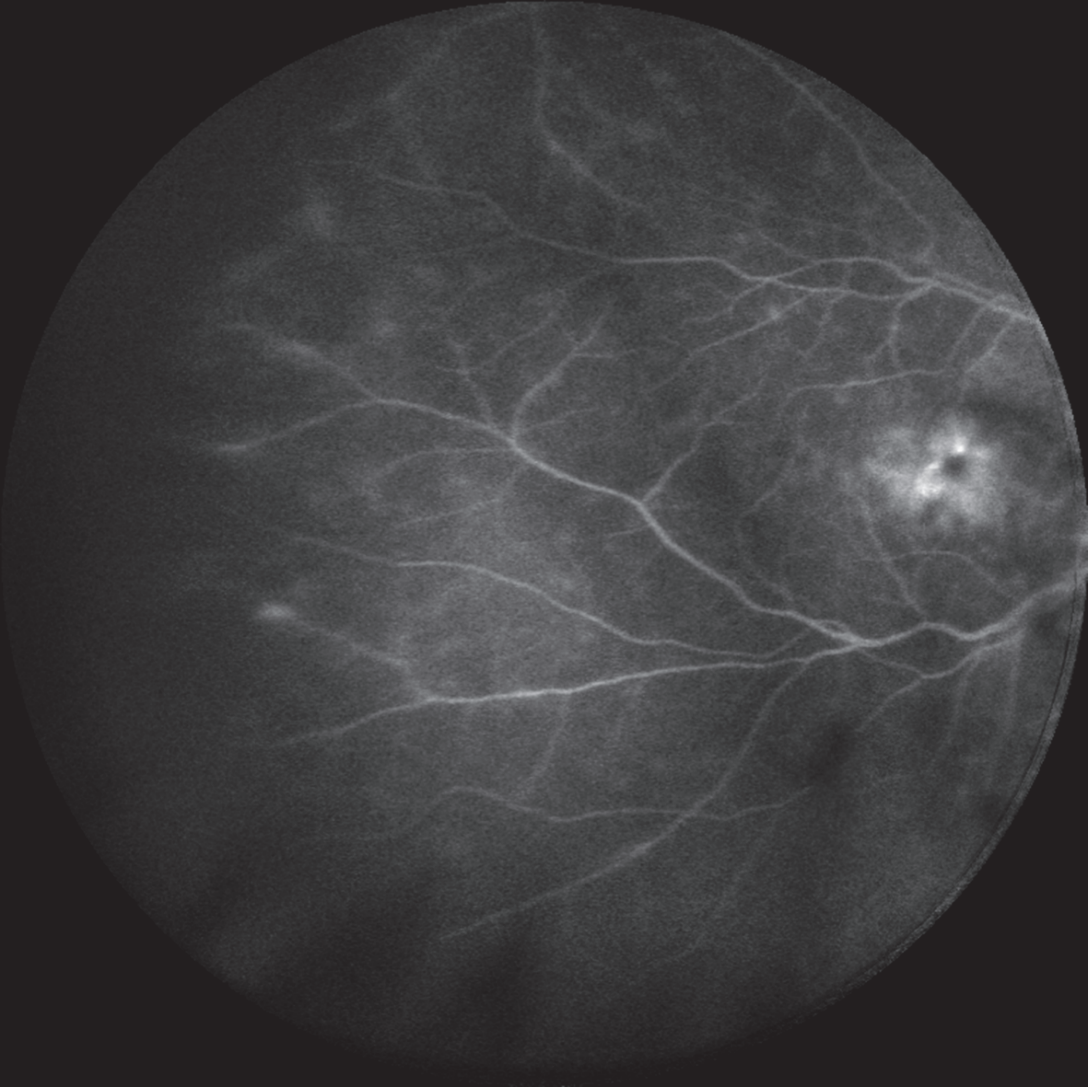
Anterior uveitis with cystoid macular edema. In a 57-year-old female with quiescent anterior uveitis, peripheral vessel leakage and cystoid macular edema were detected.

Peripheral vessel leakage was detected in 27 eyes (42%) with anterior uveitis, of which 15 eyes (55%) displayed active inflammation (Fig. 1). In eyes with an inactive stage of inflammation, peripheral vessel leakage was found in 12 eyes (31%) (p≤0.05 compared to those with active inflammation) (Fig. 2). The time from discontinuation of drug treatment and evaluation ranged from 1 month to 6 months (mean, 2.7 months). Cystoid macular edema was found in eight eyes (five with active disease and three with quiescent disease), of which seven eyes had peripheral vessel leakage (Fig. 3). Cystoid macular edema was detected in 7 of 27 eyes (26%) with peripheral vessel leakage, whereas 1 of 38 eyes (3%) did not display peripheral vessel leakage (p<0.01). In addition, peripheral retinal hyperfluorescence dots were detected in one eye.

Etiologies were identified in eight patients with anterior uveitis, with three patients diagnosed with ankylosing spondylitis, one with Fuchs heterochromic cyclitis, two with juvenile idiopathic arthritis-associated uveitis, one with tubulointerstitial nephritis uveitis, and one with sarcoid uveitis. Among these patients, peripheral vessel leakage was detected in 6 patients (12 eyes), including 3 patients with ankylosing spondylitis (with active inflammation), 1 patient with juvenile idiopathic arthritis-associated uveitis (with active inflammation), 1 patient with tubulointerstitial nephritis uveitis (with inactive inflammation, 6 months after discontinuation of drug treatment), and 1 patient with sarcoid uveitis (with inactive inflammation, 1 months after discontinuation of drug treatment). Therefore, 44.4% of the patients with peripheral vessel leakage had a specific etiology.

Based on clinical examinations and conventional fluorescein angiography, topical glucocorticoid application was intended in all of the patients with active inflammation. Because of the results of the additional ultra-wide-field angiography, 12 patients with peripheral vessel leakage and a quiescent anterior segment were added to those receiving topical glucocorticoids, while 3 patients with serious peripheral vessel leakage and an active anterior segment received a sub-Tenon injection of triamcinolone acetonide.

## Discussion

Ultra-wide-field fluorescein angiography is an important clinical method to examine the peripheral retinal area. A number of studies reported that ultra-wide-field fluorescein angiography had demonstrated the presence of new peripheral pathology in many diseases, which could not have been detected through other clinical examinations and conventional fluorescein angiography[21].

The anterior uveitis findings of the present study are of great importance. To our knowledge, the application of ultra-wide-field fluorescein angiography in anterior uveitis has not been previously reported. In the present study, 42% of the patients were found to have peripheral vessel leakage by ultra-wide-field fluorescein angiography. Clinical examinations and conventional fluorescein angiography supported the diagnosis of anterior uveitis, while ultra-wide-field fluorescein angiography additionally demonstrated peripheral vessel leakage. This could bring the diagnosis of anterior uveitis into question. In cases where the diagnosis is altered, given these findings, ultra-wide-field fluorescein angiography may be combined with other examinations to differentiate intermediate from anterior uveitis, such that the incidence rates of both would be altered. However, the clinical examination and conventional fluorescein angiography of the patients in the present study did not satisfy the standard diagnostic criteria for intermediate uveitis. Therefore, we had insufficient evidence to change the diagnosis solely based on ultra-wide-field angiography. However, we are of the view that anterior uveitis could involve peripheral vessel leakage, suggesting that inflammation in anterior uveitis includes peripheral retinal vessels in addition to the iris and ciliary body. Therefore, we propose that our findings indicate the existence of peripheral vessel leakage in anterior uveitis. Peripheral vessel leakage was also observed in 31% of the patients with quiescent disease, which suggests possible incomplete quiescence of the inflammation.

Before the adoption of ultra-wide-field fluorescein angiography, peripheral vessel leakage had gone undetected in anterior uveitis. Therefore, the possibility of a high incidence of peripheral vessel leakage in patients with anterior uveitis should be investigated. Interestingly, 44.4% of the patients with peripheral vessel leakage had specific etiologies, including ankylosing spondylitis, juvenile idiopathic arthritis-associated uveitis, tubulointerstitial nephritis uveitis, and sarcoid uveitis. In particular, all three patients with ankylosing spondylitis displayed peripheral vessel leakage. Therefore, it appeared that uveitis with a specific etiology was more likely to be associated with peripheral vessel leakage compared to idiopathic anterior uveitis. Peripheral vessel leakage was detected in 88% of the eyes with cystoid macular edema, suggesting a possible relationship between them.

As a result of ultra-wide-field angiography, a further 12 patients with peripheral vessel leakage and a quiescent anterior segment received topical glucocorticoid treatment, while 3 patients with serious peripheral vessel leakage and an active anterior segment were given a sub-Tenon injection of triamcinolone acetonide. In the present study, the final treatment strategy was based on the results of the ultra-wide-field angiography, and then compared with the previous treatment strategy arising from the examinations and conventional angiography. However, because of the lack of a control group undergoing only conventional fluorescein angiography and a relevant follow-up observation, we cannot stringently assess the outcome of the present study. Therefore, further investigation is required to evaluate the efficacy of the treatment.

Most of the previous studies used the Optos system, whereas we used the Heidelberg Spectralis. These systems differ in several ways. The ultra-wide-field Heidelberg Spectralis module can image the superior and inferior retina more peripherally than the Optos, whereas the Optos is more effective in the temporal and nasal retina. Lid and lash artifacts are observed less frequently in the Heidelberg Spectralis than in the Optos. Image distortion appears less in the Heidelberg Spectralis noncontact lens than in the Optos system. The Heidelberg ultra-wide-field lens obtains better quality images than the Optos, particularly in the superior and inferior quadrants[22].

The present study involved several limitations that restricted the interpretation of the data. The ultra-wide-field module of the Heidelberg Spectralis HRA was unable to obtain a single image of the whole retina. We only defined the patients as active or quiescence, and thus were unable to evaluate the relationship between the severity of the inflammation and peripheral vessel leakage. We acquired the activity and management data based on the clinical examination and conventional fluorescein angiography prior to the ultra-wide imaging of the patient. There was a potential selection bias in the study population, including a possible tendency for the selection of patients who were more likely to have peripheral retinal findings. While the angiographic results prompted us to change our treatment strategy, we did not know whether this was beneficial. Consequently, there may have been excessive treatment in some cases compared to that which was actually required for an adequate clinical response.

In conclusion, the results suggest that ultra-wide-field fluorescein angiography is very useful in detecting peripheral retinal pathology in anterior uveitis, including peripheral retinal vessel leakage, which is an important manifestation of uveitis. The detection of such vascular changes may alter the assessment of disease activity and management decisions.
